# Dietary and non-dietary determinants of linear growth status of infants and young children in Ethiopia: Hierarchical regression analysis

**DOI:** 10.1371/journal.pone.0209220

**Published:** 2019-01-25

**Authors:** Shimels Hussien Mohammed, Tesfa Dejenie Habtewold, Balewgizie Sileshi Tegegne, Mulugeta Molla Birhanu, Tesfamichael Awoke Sissay, Bagher Larijani, Ahmad Esmaillzadeh

**Affiliations:** 1 Department of Community Nutrition, School of Nutritional Sciences and Dietetics, Tehran University of Medical Sciences-International Campus (TUMS-IC), Tehran, Iran; 2 Department of Epidemiology, University Medical Center Groningen, University of Groningen, Groningen, The Netherlands; 3 School of Nursing, College of Health Sciences, Mekelle University, Mekelle, Ethiopia; 4 Endocrinology and Metabolism Research Center, Endocrinology and Metabolism Clinical Sciences Institute, Tehran University of Medical Sciences, Tehran, Iran; 5 Obesity and Eating Habits Research Center, Endocrinology and Metabolism Molecular Cellular Sciences Institute, Tehran University of Medical Sciences, Tehran, Iran; 6 Department of Community Nutrition, School of Nutritional Sciences and Dietetics, Tehran University of Medical Sciences, Tehran, Iran; 7 Food Security Research Center, Department of Community Nutrition, Isfahan University of Medical Sciences, Isfahan, Iran; Harvard TH Chan School of Public Health, UNITED STATES

## Abstract

**Introduction:**

Childhood growth faltering remains a major public health problem in developing countries. We aimed to identify the distal, underlying, and proximal dietary and non-dietary factors associated with length-for-age (LFA) of infants and young children in Ethiopia.

**Methods:**

We used a nationally representative sample of 2,932 children aged 6–23 months from the Ethiopian demographic and health survey (EDHS) conducted in 2016. Hierarchical regression analysis was done to identify the factors associated with LFA.

**Findings:**

Pastoral residence (adjusted β (aβ) = -0.56, 95%CI = -0.82, -0.31, P<0.001) and poorest household wealth category (aβ = -0.57, 95%CI = -0.66, -0.48, P<0.001) were the basic factors negatively associated with LFA. Among underlying factors, maternal wasting (aβ = -0.43, 95%CI = -0.58, -0.28, P<0.001), and unimproved toilet facility (aβ = -0.48, 95%CI = -0.73, -0.23, P<0.001) were negatively associated with LFA. Proximal factors found positively associated with LFA were dietary diversity (aβ = 0.09, 95%CI = 0.043, 0.136, P<0.001), meal frequency (aβ = 0.04, 95%CI = 0.00, 0.08, P = 0.042), and vitamin A supplementation (aβ = 0.16, 95%CI = 0.03, 0.29, P = 0.020). Male sex (aβ = -0.26, 95%CI = -0.39, -0.14, P<0.001), age (aβ = -0.12, 95%CI = -0.13, -0.10, P = 0.001), small birth size (aβ = -0.45, 95%CI = -0.62, -0.29, P<0.001), and not currently breastfeeding (aβ = -0.29, 95%CI = -0.47, -0.11, P = 0.003) were negatively associated with LFA.

**Conclusion:**

LFA was associated with various influences at distal, underlying, and proximal levels. A multi-pronged approach, addressing the various factors comprehensively, would represent an important consideration to promote linear growth in early childhood in Ethiopia.

## Introduction

Stunting, an indicator of linear growth failure, poses a significant public health challenge in developing countries [1]. Achieving healthy child growth has taken a central focus in global nutrition programs. The World Health Organization (WHO) prioritized stunting reduction as a priority nutrition target for the period 2012–2015 and aimed for a two-fifths decline in the prevalence of stunting by 2025, from the figure in 2010 [2]. One of the highest global stunting burdens has been in Ethiopia, where 44% and 38% of children under 5 years of age were stunted in the years 2011 and 2016 [3], respectively. While the last two decades have seen a reduction in the proportion of stunting in Ethiopia, the rate of decline has been below the international goal [3].

Linear growth failure is a multifactorial problem, with risk factors originating from various levels and often interacting, or correlating, with each other. In the United Nations International Children's Emergency Fund (UNICEF) conceptual framework of malnutrition, contributors to undernutrition were classified into basic, underlying, and immediate (proximal) risk factors [4]. The immediate and underlying influences often include suboptimal infant and young child feeding practices (IYCFP), poor sanitation and hygiene, and childhood infection. Poor socioeconomic status is a basic determinant for most forms of under-nutrition [2, 4, 5].

In Ethiopia, most of the existing evidence on correlates of linear growth is derived from studies on the determinants of stunting [6–8], as defined by length-for-age (LFA) <-2 Z-score [1, 9]. Notwithstanding its widespread use and programmatic relevance, it could, however, be acknowledged that the approach would misclassify children with mild stunting (-2≤ LFA <-1 Z-scores) as not-stunted [10]. Another methodological limitation of most of the existing studies on determinants of LFA is that their analyses did not account for the hierarchical nature and interrelationships among the multilevel growth influences. For example, the basic determinants of LFA influence not only LFA directly but also its proximal or underlying determinants [4]. Thus, including all variables in one model, a practice in most of the existing studies, may nullify or weaken the association of distal factors with the outcome [11]. This study was done addressing these limitations, such that we used LFA on a continuous scale and conducted hierarchical regression analyses which took into account the hierarchical nature the determinants of LFA. The various predictors of linear growth status depicted in the UNICEF conceptual framework of child malnutrition causation (distal, underlying, and proximal dietary as well as non-dietary factors) were examined [4]. The interrelationship among these predictor variables was also considered. The literature recommends adopting hierarchical regression approaches to multiple exposure studies, rather than forcing all exposure variables in one model [11, 12]. Thus, we followed three-stage hierarchical regression approaches in examining the relation of the exposure variables with the outcome variable (linear growth status).

## Methods

### Data source

We used the data of children included in EDHS 2016 [3]. The survey was part of the international demographic and health survey (DHS) program, run primarily by the United States Agency for International Development [13]. The full data set of EDHS 2016 is available on the DHS program website: http://dhsprogram.com/data/dataset/Ethiopia_Standard-DHS_2016.cfm.

### Sampling methodology and sample size

Study participants’ recruitment followed a stratified, two-stage cluster sampling. Administratively, Ethiopia is divided into nine regions and two administrative cities. Each region was stratified into urban and rural areas, yielding 21 sampling strata. In the first stage, census enumeration areas (CEAs) were used as primary sampling units. A total of 645 CEAs were selected, with probability proportional to size method. In the second stage of sampling, a fixed number of 28 households per cluster were selected with systematic random sampling. The final sample included a total of 16,650 households. All children under 5 years of age were considered eligible for the survey [3]. As most of the growth faltering occurs in the first two years after birth [14], we extracted only the data set of children aged 6–23 months. The lack of data on IYCFP of those above 24 months in the dataset was another reason to restrict the study to the age group 6–23 months. We found a total of 3,005 children aged 6–23 months in the dataset. Of these, 359 with missing data were excluded. We further applied weighting on the remaining 2,746 children, resulting in a final sample of 2,932 children (weighted). The weighting was applied to compensate for the unequal probability of selection by regions and ensure the sample resembled the national population structure. We followed the DHS sample weighing approach [3].

### Variables and measurements

#### Outcome variable

The outcome variable of the study was measured by LFA. Length of children was measured in lying position. Age of child (in months) was obtained from the parents [3]. Using the length and age data, LFA Z-score was calculated for each child using the WHO 2006 Child Growth Standards [9]. As LFA is a reflection a child's linear growth status [9], it is used in this report interchangeably with linear growth status. We used LFA Z-scores on a continuous scale in all analyses.

#### Explanatory variables

**Fig 1:**
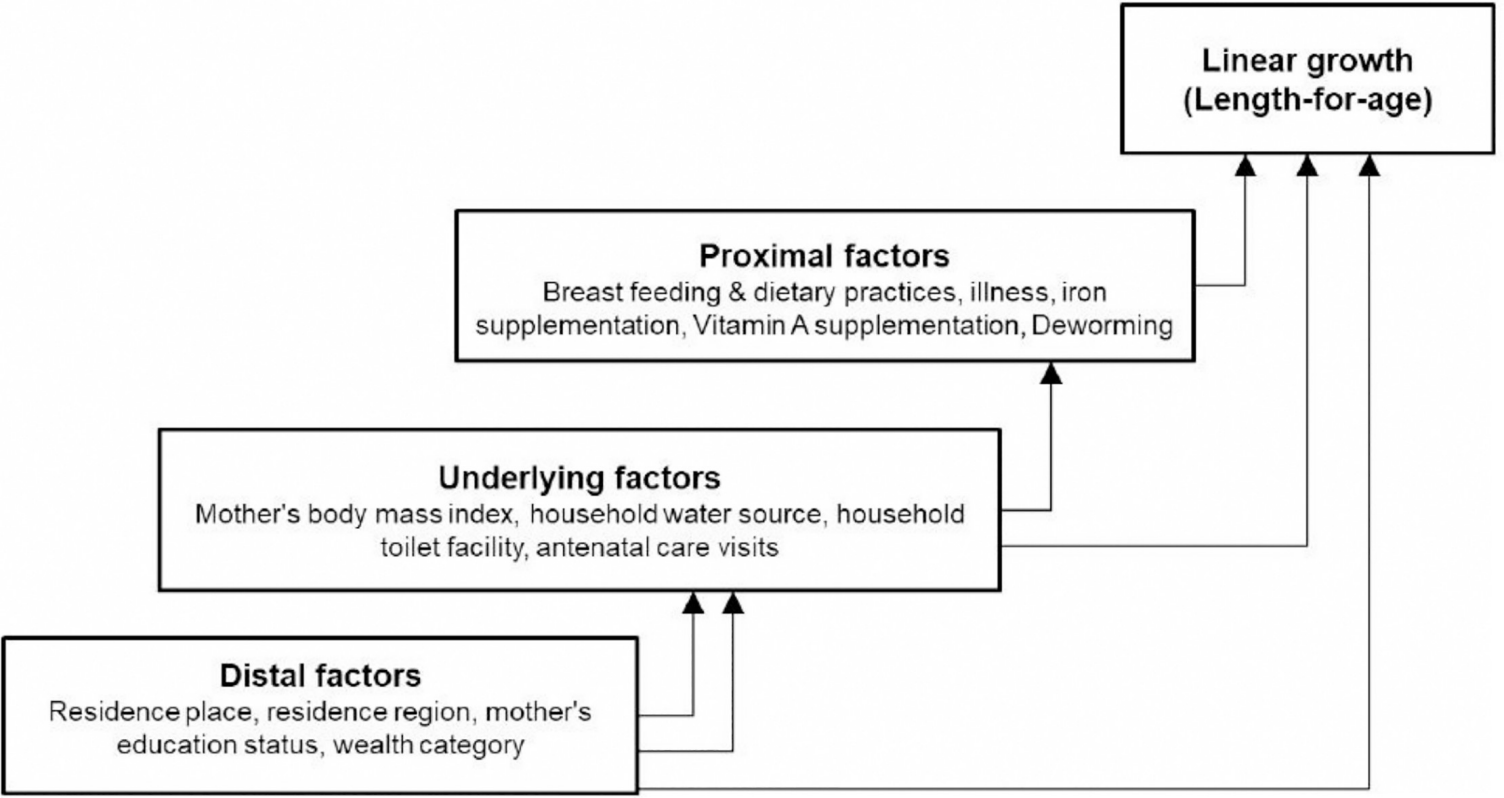
Conceptual framework of factors influencing linear growth status.

We selected the predictor variables guided by the literature, the UNICEF conceptual framework of malnutrition causation, and availability of the variable in the dataset. The variables were categorized into three groups: basic, underlying, and proximal factors. A conceptual framework of the variables influencing LFA is shown in Fig 1 and described as follows:

1. Basic (distal) factors: Residence place (urban, rural), residence region ('mainly agrarian' and 'mainly pastoral'), mother’s education status (no education, primary, secondary and above), and household wealth category (poorest, poorer, middle, richer, and richest). Regions classified as ‘mainly agrarian’ were Amhara, Oromia, Tigray, South, and Addis Ababa. Regions classified as ‘mainly pastoral’ were Somali, Afar, Harari, Gambella, Benishangul-Gumuz, and Dire Dawa. The household wealth category was determined by principal component analysis using the asset variables collected during the survey.
2. Underlying factors: Source of drinking water (improved, unimproved), toilet facility (improved, unimproved), mother’s body mass index (BMI) in Kg/m^2^ (<18.5, ≥18.5), and antenatal care visits (ANC). Improved sources of drinking water included piped water, bottled water, and protected wells in the compound. Unprotected wells, springs, rivers, ponds, lakes, and dams were grouped as unimproved water sources. Improved household toilet facilities referred to flush toilets or ventilated pit latrines. Traditional pit latrines were considered as unimproved facilities [3]. ANC visits referred to the number of health facility visits the mother attended during pregnancy of the indexed child and categorized into two groups (<4, ≥4).
3. Proximal factors: Sex, age (months, obtained from child health cards or parents), birth type (single, twin), birth size (as reported subjectively by the mother of the child, categorized into three groups categories: small, average, and large), health status and IYCFP. History of infection (yes, no; assessed by whether the child had had cough, diarrhea, or fever in the two weeks prior to the survey). Current breastfeeding status (yes, no), initiation of breastfeeding within the first one hour after birth (yes, no), deworming in last six months (yes, no), and vitamin A supplementation in last six months (yes, no), history of iron supplement use (ISU) in the last seven days (yes, no), meal frequency, and dietary diversity were also assessed. Scoring of dietary diversity and meal frequency was developed using the 24 hours dietary recall data and following the IYCFP guidelines [15] and DHS methodology [13].

### Statistical analysis

All analyses were done on the weighted data and taking into account the cluster design of the study. The weighting was done to compensate for the unequal probability of sample selection across the strata (regions) [3, 13]. The cluster adjustment was applied to account for the variance inflation effect of the cluster sampling. Statistical assumptions, like normality of LFA distribution and collinearity among continuous explanatory variables, were checked. The relation of each explanatory factor with LFA was first assessed by bivariable analyses. Then, we conducted a hierarchical regression analysis using the variables with P-value <0.25 in the bi-variable analyses. The hierarchical regression was done considering the hierarchical nature of the relationship among the predictor variables, shown in the conceptual framework (Fig 1). Thus, three models were constructed. In the first model, we included the basic factors as they were the ones less likely to be affected by the other variables. In the second model, we included the underlying (intermediate) factors and factors from model 1 with P<0.25. In the last model, model 3, we included the proximal factors and factors from model 2 with P<0.25. Statistical significance (P≤0.05) of each explanatory variable in the hierarchical regression analysis was determined at the corresponding model in which the variable was first entered, irrespective of the performance of the variable in the subsequent model(s). This approach was aimed to avoid the possibility of intermediate variables removing, or weakening, the association of the distal factors with the outcome variable [11]. All data analyses were conducted with STATA version 14.

### Ethical consideration

This work was based on a publicly available dataset, EDHS 2016. The original survey protocol was approved by the Intuitional Review Boards (IRB) of Ethiopian Public Health Institute, Ethiopian National Research Ethics Review Committee, and ICF International [3]. Oral consent was taken from study participants before data collection. For this particular work, we obtained additional ethical approval from IRB of Tehran University of Medical Sciences, ethical code IR.TUMS.VCR.REC.1397.142, and approval to use the dataset from the DHS program through a project titled “trends and determinants of malnutrition in Ethiopia." The data was used solely for the purpose requested.

## Results

**Fig 2:**
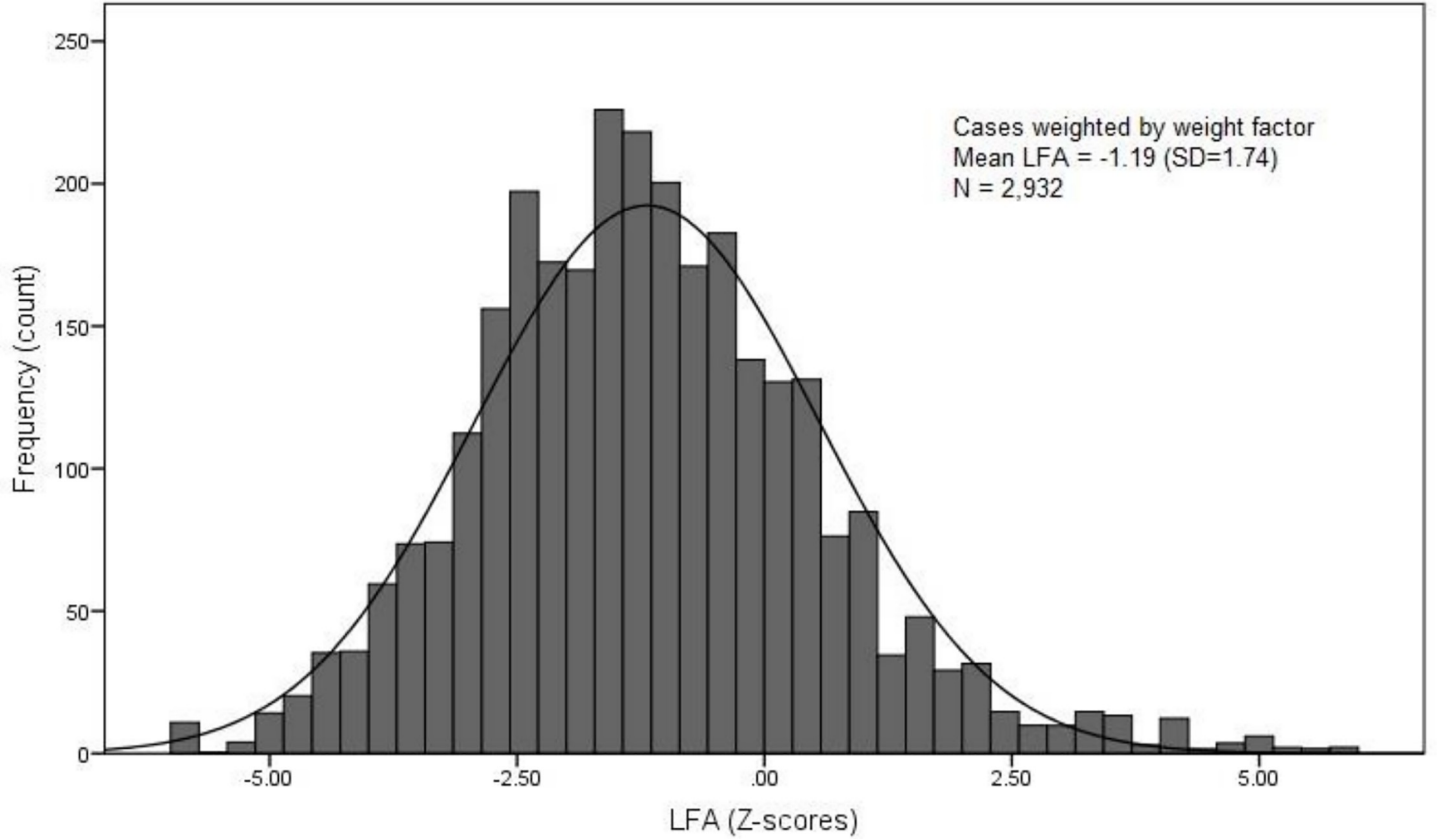
Length-for-age distribution of study participants (N = 2,932).

We included a total of 2,932 (weighted) children aged 6–23 months, 1,371 (46.76%) boys and 1,561 (53.24%) girls. The majority of children were from rural areas (88.35%). The mean age (SD) of participants was 14.0 (5.13) months. The majority of children were from middle- and low- income households (67.51%). The mean LFA (SD) of the participants was -1.19 (1.74) Z-scores. The LFA (Z-score) distribution of all the children included in this work is shown in Fig 2. The graph shows a normally distributed LFA at its mean (LFA = -1.74). However, it is shifted to the left of the Z-score = 0 line, indicating a population with growth faltering (stunting). Overall, 32.73% of the children were stunted (LFA < -2 Z-score).

Table 1 presents the results of the bivariable analyses on the relationship of the basic (distal) and underlying factors with LFA. Rural residence was associated with lower mean LFA (P<0.001). Children from 'mainly agrarian' regions were significantly more stunted than children from 'mainly pastoral' regions (P = 0.001). Poor household wealth category and low maternal education status were also associated with a significantly lower mean LFA (P<0.001). Of the underlying factors, lack of improved toilet facility (P<0.001) and ANC<4 during pregnancy (P = 0.003) were significantly associated with lower LFA.

**Table 1:** Relation of basic and underlying factors with length-for-age (N = 2,932).

Variables	Weighted frequency (%)	Mean LFA (95% CI)	P*
**Basic factors**			
Residence place			
Urban	11.65	-0.85 (-1.01, -0.68)	<0.001
Rural	88.35	-1.23 (-1.30, -1.16)	
Residence region			
Pastoral (mainly)	6.69	-0.78 (-1.05, -0.51)	0.001
Agrarian (mainly)	93.31	-1.22 (-1.28, -1.15)	
Household wealth category			
Poorest	23.92	-1.58 (-1.73, -1.43)	<0.001
Poorer	22.88	-1.30 (-1.44, -1.16)	
Middle	20.71	-1.06 (-1.20, -0.95)	
Richer	18.09	-0.98 (-1.12, -0.83)	
Richest	14.40	-0.93 (-1.04, -0.82)	
Maternal education status			
Illiterate	60.94	-1.26 (-1.34, -1.18)	<0.001
Primary	30.82	-1.18 (-1.29, -1.07)	
Secondary+	8.24	-0.65 (-0.82, -0.47)	
**Underlying factors**			
Maternal BMI (kg/m^2^)			
<18.5	24.07	-1.56 (-1.68, -1.44)	<0.001
18.5+	75.93	-1.08 (-1.15, -1.00)	
Water source			
Not improved	42.76	-1.22 (-1.32, -1.12)	0.407
Improved	57.24	-1.16 (-1.25, -1.08)	
Toilet facility			
Not improved	90.56	-1.26 (-1.33, -1.19)	<0.001
Improved	9.44	-0.48 (-0.66, -0.30)	
Antenatal care visits			
<4	65.67	-1.25 (-1.33, -1.17)	0.003
4+	34.33	-1.05 (-1.15, -0.94)	

LFA: Length-for-age; CI: Confidence Interval; BMI: Body mass index.

*P: Based on one-way ANOVA test of association.

The results of bi-variable analyses between the proximal determinants and LFA are shown in Table 2. Boys were significantly more stunted than girls (P = 0.005). Child age, current breastfeeding, dietary diversity, and meal frequency were significantly associated with LFA (P<0.05). Birth type (being twin) and small birth size were significantly associated with lower mean LFA (P<0.001). Children who received vitamin A supplement in the six months before the survey had a significantly higher mean LFA compared with those who did not receive it (P = 0.005). History of infection, early initiation of breastfeeding, use of deworming medication in the previous six months, and ISU in the previous seven days were not significantly associated with LFA (P>0.05).

**Table 2:** Relation of proximal factors with length-for-age (N = 2,932).

Variables	Weighted frequency (%)	Mean LFA (95% CI)	P*
Child sex			
Boy	46.76	-1.28 (-1.38, -1.19)	0.005
Girl	53.24	-1.10 (-1.19, -1.02)	
Age (months)	100.00	-1.19 (-1.25, -1.12)	<0.001
Birth type			
Single	97.35	-1.17 (-1.23, -1.10)	<0.001
Twin	2.65	-1.88 (-2.14, -1.62)	
Birth size			
Small	27.68	-1.48 (-1.61, -1.36)	<0.001
Average	40.69	-1.20 (-1.30, -1.11)	
Large	31.64	-0.91 (-1.02, -0.79)	
Infection (in last 2 weeks)^a^			
No	74.48	-1.15 (-1.23, -1.07)	0.203
Yes	25.52	-1.25 (-1.37, -1.12)	
Current breastfeeding status			
No	10.16	-1.74 (-2.02, -1.46)	<0.001
Yes	89.84	-1.13 (-1.25, -1.01)	
Early initiation of breastfeeding			
No	10.74	-1.15 (-1.36, -0.94)	0.858
Yes	89.26	-1.17 (-1.24, -1.09)	
Deworming (in last 6 months)			
No	90.88	-1.20 (-1.26, -1.13)	0.395
Yes	9.12	-1.10 (-1.31, -0.89)	
Vitamin A supplement (in last 6 months)			
No	56.39	-1.12 (-1.20, -1.03)	0.005
Yes	43.61	-1.30 (-1.39, -1.20)	
Iron supplement (in last 7 days)			
No	92.10	-1.20 (-1.27, -1.13)	0.356
Yes	7.90	-1.09 (-1.29, -0.88)	
Meal frequency^b^	100.00	-1.19 (-1.25, -1.12)	0.001
Dietary diversity^c^	100.00	-1.19 (-1.25, -1.12)	0.003

LFA, Length-for-age; CI, Confidence interval.

^a^ = Infection defined as history of cough, diarrhea or fever in the last 2 weeks preceding the survey (yes, any one of the three conditions).

^b^ = Meal frequency defined as, according to the WHO criteria, when a child ate at least 3 and 4 times a day for breastfeeding and non-breastfeeding, respectively.

^c^ = Dietary diversity defined as, according to the WHO criteria eating from 4 or more of the 7 food groups: (i) flesh foods, (ii) eggs, (iii) dairy products, (iv) grains, roots, and tubers, (v) legumes and nuts, (vi) vitamin-A rich fruits and vegetables, and (vii) other fruits and vegetables.

*P: Based on one-way ANOVA tests for categorical variables or Pearson’s correlation tests for continuous variables.

Table 3 presents the results of the hierarchical regression analyses. After adjusting for co-variate factors, shown in model 1 of Table 3, the basic factors found significantly associated with LFA were region and household wealth category. Children in agrarian regions were significantly more stunted than children in pastoral regions (aβ = -0.56, 95%CI = -0.82, -0.31, P<0.001). The mean LFA of children from poorest, poorer, and middle households was significantly lower that of children from richest households (P<0.001). Among the underlying factors, type of household toilet facility (aβ = -0.48, 95%CI = -0.73, -0.23, P<0.001) and mother’s BMI (aβ = -0.43, 95%CI = -0.58, -0.28, P<0.001) were significantly associated with LFA. Children living in households with unimproved toilet facilities were more stunted, with their LFA lower by 0.48 Z-scores, compared with those living in households with improved toilet facilities (aβ = -0.48, 95% CI = -0.73, -0.23, P<0.001). Children of wasted mothers (BMI <18.5 kg/m^2^) had a significantly lower mean LFA than children of non-wasted mothers (aβ = -0.43, 95%CI = -0.58, -0.28, P<0.001). ANC follow-up <4 times was not significantly associated with lower mean LFA (aβ = -0.02, 95%CI = -0.09, -0.05, P = 0.650).

**Table 3:** Hierarchical regression analysis of the relation of basic, underlying, and proximal factors with length-for-age.

Variables	Model 1^a^		Model 2^b^		Model 3^c^	
	Adjusted β (95% CI)	P	Adjusted β (95% CI)	P	Adjusted β (95% CI)	P
Residence place						
Rural	-0.02 (-0.26, 0.23)	0.881				
Urban	Reference					
Residence region						
Pastoral (mainly)	Reference					
Agrarian (mainly)	-0.56 (-0.82, -0.31)	<0.001*				
Wealth category						
Poorest	-0.57 (-0.66, -0.48)	<0.001*				
Poorer	-0.31 (-0.39, -0.22)	<0.001*				
Middle	-0.12 (-0.18, -0.06)	0.006*				
Richer	-0.02 (-0.18, 0.14)	0.801				
Richest	Reference					
Maternal education status						
No education	-0.44 (-0.90, 0.03)	0.068				
Primary	-0.41 (-0.87, 0.06)	0.087				
Secondary+	Reference					
Toilet facility						
Unimproved			-0.48 (-0.73, -0.23)	<0.001*		
Improved			Reference			
Maternal BMI (kg/m^2^)						
<18.5			-0.43 (-0.58, -0.28)	<0.001*		
≥18.5			Reference			
Antenatal care visits						
<4			-0.02 (-0.09, -0.05)	0.650		
≥4			Reference			
Child sex						
Boy					-0.26 (-0.39, -0.14)	<0.001*
Girl					Reference	
Child age (in months)					-0.12 (-0.13, -0.10)	<0.001*
Birth type						
Twins					-0.21 (-0.84, 0.42)	0.514
Single					Reference	
Birth size						
Small					-0.45 (-0.62, -0.29)	<0.001*
Average					-0.24(-0.39, -0.09)	0.002*
Large					Reference	
Infection (in last 2 weeks)^d^						
No					Reference	
Yes					-0.08(-0.22, 0.06)	0.493
Current breastfeeding status						
No					-0.29 (-0.47, -0.11)	0.003
Yes					Reference	
Meal frequency^e^					0.04 (0.001, 0.078)	0.042*
Dietary diversity^f^					0.09 (0.043, 0.136)	<0.001*
Vitamin A supplement (in last 6 months)						
No					Reference	
Yes					0.16 (0.03, 0.29)	0.020*
Model summary						
Explained variance [R^2^ (%)]	31.74		53.58		61.47	

CI, Confidence interval; BMI, Body mass index.

^a^Model 1: adjusted for residence place, residence region, wealth category, maternal age, and maternal education.

^b^Model 2: adjusted for residence region, wealth category, maternal age and maternal education, toilet facility, maternal BMI, and antenatal care.

^c^Model 3: residence region, wealth category, maternal age and maternal education, toilet facility, maternal BMI, child sex, child age, birth type, birth size, infection, current breastfeeding, minimum meal frequency, minimum meal diversity, and vitamin A supplement.

^d^Infection defined as history of cough, diarrhea or fever in the last 2 weeks preceding the survey (yes, any one of the three conditions).

^e^Meal frequency defined as, according to the WHO criteria, when a child ate at least 3 and 4 times a day for breastfeeding and non-breastfeeding, respectively.

^f^Dietary diversity defined as, according to the WHO criteria eating from 4 or more of the 7 food groups: (i) flesh foods, (ii) eggs, (iii) dairy products, (iv) grains, roots, and tubers, (v) legumes and nuts, (vi) vitamin-A rich fruits and vegetables, and (vii) other fruits and vegetables.

*Significant at P<0.05.

Of the proximal factors (Model 3, Table 3), child's sex, age, birth size, current breastfeeding status, meal frequency, dietary diversity, and vitamin A supplementation were significantly associated with LFA after adjusting for covariate factors. Mean LFA was significantly lower in boys, compared with girls (aβ = -0.26, 95%CI = -0.39, -0.14, P<0.001). A month increase in a child’s age was associated with 0.12 Z-scores LFA deficit (aβ = -0.12, 95%CI = -0.13, -0.10, P = 0.001). Compared with large birth size, small birth size was associated with 0.45 Z-scores deficit in LFA (aβ = -0.45, 95% CI = -0.62, -0.29, P<0.001) and average birth size with 0.24 Z-scores deficit in LFA (aβ = -0.24, 95% CI = -0.39, -0.09, P = 0.002). Mean LFA was significantly lower in children who were not on breastfeeding, compared with those on breastfeeding (aβ = -0.29, 95% CI = -0.47, -0.11, P = 0.003).

A unit increase in frequency of meal was associated with 0.04 Z-scores increase in LFA (aβ = 0.04, 95%CI = 0.001, 0.078, P = 0.042). A unit increase in dietary diversity was associated with 0.09 Z-scores increase in LFA (aβ = 0.09, 95%CI = 0.043, 0.136, P<0.001). Vitamin A supplement use in the previous six months was associated with a significantly higher mean LFA (aβ = 0.16, 95%CI = 0.03, 0.29, P = 0.020). The mean LFA of children with history of infection was not significantly different from that of children with no history of infection (aβ = -0.08, 95%CI = -0.22, 0.06, P = 0.493). The mean LFA of twins was also not significantly different from that of single births (aβ = -0.21, 95%CI = -0.84, 0.42, P = 0.514).

## Discussion

The factors that demonstrated significant negative associations with LFA were agrarian region of residence, poor household wealth category, unimproved household toilet facility, male sex, age, not currently breastfeeding, and small birth size. Vitamin A supplement use, higher meal frequency, and dietary diversity scores showed significant positive associations with LFA.

There was a significant discrepancy in LFA by residence region, such that LFA was significantly lower in children living in agrarian regions than those living in pastoral regions. The finding was consistent with reports of previous studies in Ethiopia [3, 6, 16]. The prevalence of stunting was high in Amhara and Tigray, both agrarian regions, but low in Somali and Afar, both pastoral regions [3, 16]. The same pattern was observed in adults’ height in Ethiopia [3, 16]. Thus, a possible explanation for the agrarian-pastoral LFA discrepancy could be intergenerational influence on height. Children of short parents may be more likely to be shorter. Height is known to be influenced by both genetic and environmental factors [17–19]. It could also be due to regional variation in dietary practices. For example, consumption of animal milk is more common in pastoral communities than in agrarian communities of Ethiopia. Milk is a good source of nutrients and other bioactive substances necessary for bone growth, including calcium. Insulin-like growth factor-I, a bone growth promoting factor, is also present in a higher concentration in animal milk than in human milk. Household wealth category was also significantly associated with LFA. Children from poor households were, on average, more stunted than children from affluent households. Poverty is an important basic determinant of child growth as well as wellbeing [4, 5]. On average, children dwelling in households with unimproved toilet facilities were more stunted than children dwelling in households with improved toilet facilities. Poor sanitation is a risk factor for childhood infection, which subsequently affects LFA negatively [5, 20, 21] Children of wasted mothers were more likely to be shorter than those of non-wasted mothers. The same finding was reported by other studies done in sub-Saharan countries [5, 22], and it could be because of possibly poor quality and quantity of breast milk of malnourished mothers. It might also be due to household food insecurity, which could negatively impact the nutritional status of the mother and the child as well.

Child age was inversely related to linear growth status. The finding is consistent with other reports [4, 5, 22]. The suboptimal child complementary feeding practices in Ethiopia could account for the phenomenon. Studies have shown a high proportion of children in Ethiopia not meeting the minimum acceptable diet [3, 23]. The impact of poor complementary feeding would be profound as the child’s age increases, during which the mother’s breast milk becomes short of fulfilling the growing demand of the child. Besides, as LFA faltering is a reflection of chronic undernutrition [1], its expression would be more evident through time [14, 24–26]. Children born with small birth size had a significantly lower LFA, a finding consistent with previous reports [5, 24]. LFA increased with an increase in dietary diversity and meal frequency scores. Vitamin A supplementation in the last six months was related to higher LFA. Improving IYCFP, as well as micronutrient supplementation, stands at the center of global child nutrition recommendations [15, 27].

In this study, ISU and deworming did not demonstrate a significant association with LFA. Iron is essential for many bodily functions, including the expression of growth-promoting factors such as insulin-like growth factor-I [28]. Deworming could also be presumed to promote linear growth, most likely by decreasing intestinal parasites or improving hemoglobin status [29]. Notwithstanding the role of ISU in promoting child health, our finding is consistent with a recent meta-analysis and empirical report [30, 31].The lack of association of LFA with ISU or deworming might be due to the fact that in DHS studies, frequency and adherence to these medications were not assessed, though it could be acknowledged that these factors influence body responses to micronutrient interventions or, alternatively, because of the multifactorial nature of iron regulation [32]. For example, given the high childhood morbidity in developing countries, serum hepcidin could be expected to be high, which might blunt the effect of the iron by decreasing iron absorption and ferroportin expression. Thus, taking ISU may not necessarily increase iron availability for erythropoiesis [32].

Our finding that LFA was related to both dietary and non-dietary factors confirms the multifactorial nature of LFA. Some of the factors, like dietary diversity, are directly related to child nutrition practices. Other factors like education status and household income could be considered to be indirectly associated with the child’s health condition. Reducing childhood linear growth faltering is one of the main targets of the Ethiopian National Nutrition Program [33]. To that end, the design and implementation of comprehensive nutrition interventions stands an important strategy. It is essential to implement both nutrition-sensitive and nutrition-specific interventions to achieve optimal child growth and reduce the burden of stunting in Ethiopia. The existing nutrition-specific interventions [33], like the promotion of optimal infant and young child feeding practices, and micronutrient supplementation, should be strengthened. The nutrition-sensitive interventions, particularly in education, health and agriculture sectors should also be strengthened as educational status, sanitation and food security are among the main determinants of child health and wellbeing. The integration and coordination of the activities of the various sectors with a stake in nutrition and public health is also as important as the design of comprehensive interventions.

One of the strengths of this study is the use of hierarchical regression analysis based on a predefined conceptual framework. The approach took into account the hierarchical nature of the determinants of LFA. Some factors, particularly the distal factors like educational status and income, affect not only LFA but also its intermediate and proximal determinants. In other words, the intermediate and the proximal factors mediate the link of the basic factors to LFA. Thus, the use of the standard multivariable analysis, i.e., entering both distal and mediator variables in the same model, is problematic because the mediator variables will weaken or nullify the true association between the distal factors and the outcome of interest. Other strengths of the study are the use of a nationally representative sample which may make the findings more generalizable to the study setting, and the inclusion of various explanatory factors which might have improved comprehensiveness of the study. On the other hand, the cross-sectional design of the study precluded making causal inferences. Thus, a definite conclusion could not be drawn on the relationships of the determinant factors with LFA. Furthermore, the collection of data on some variables based on respondents’ memory of past events might have introduced recall bias.

## Conclusion

LFA was associated with various dietary and non-dietary influences, indicating the multifactorial nature of linear growth status of infants and young children. A comprehensive approach would stand an important consideration for promotion of optimal child linear growth in Ethiopia.
